# Glass Drainage Tubes in Prostatic Surgery

**Published:** 1909-03

**Authors:** Follen Cabot

**Affiliations:** New York


### GLASS DRAINAGE TUBES IN PROSTATIC SURGERY.

BY FOLLEN CABOT, M. D., NEW YORK.

The problem of drainage in surgery of the prostate is a very important one. Many methods of draining the bladder after prostatectomy have been employed.

I have never been satisfied with rubber drainage tubes. They are difficult to keep in place, and are generally, to my mind, unsatisfactory. The more closely we can observe prostatic cases the better; therefore it occurred to me that glass could be used to great advantage for this purpose. The tube I am about to describe is one made for me by Tieman & Co. I here refer to tubes for use in suprapubic work.

The tube is a double current glass tube of about 35 French scale bent at a right angle. The bladder end is about four inches long and has a large eye near the end. Running up from the eye on the outside is a small glass tube made as a part of the large one. This follows the large tube straight up and does not curve. It projects an inch or trifle more above the right angle turn. The outer part of larger tube is 2 1-2 inches long, and points towards the patient's pubic region. This double flow tube may be held in place by adhesive plaster or bandage with hole cut for entrance of small tube.

On each outlet we place rubber tubing, and if we so desire, we can have a constant flow of solution in at small tube, out at large one. The flow can be closely watched. The tube is always clean and held securely in place. The distance it projects into the bladder may be increased or decreased by padding of gauze under external arm of tube.

It is well to have two or three sizes of these tubes.

In the two steps operation, preliminary cystotomy and secondary prostatectomy, we can very easily find the best position for the tube after the cystotomy and continue it after the prostatectomy.

I have used these tubes in several cases and they have been very much more satisfactory than any I have ever tried before.

New York, 129 E. 31st street.

